# Disparities in Care Management During Terminal Hospitalization Among Adults With Metastatic Cancer From 2010 to 2017

**DOI:** 10.1001/jamanetworkopen.2021.25328

**Published:** 2021-09-22

**Authors:** Stephanie Deeb, Fumiko L. Chino, Lisa C. Diamond, Anna Tao, Abraham Aragones, Armin Shahrokni, Divya Yerramilli, Erin F. Gillespie, C. Jillian Tsai

**Affiliations:** 1 Icahn School of Medicine at Mount Sinai, New York, New York; 2 Department of Radiation Oncology, Memorial Sloan Kettering Cancer Center, New York, New York; 3 Immigrant Health and Cancer Disparities Service, Memorial Sloan Kettering Cancer Center, New York, New York; 4 Tufts University School of Medicine, Boston, Massachusetts; 5 Department of Psychiatry and Behavioral Sciences, Memorial Sloan Kettering Cancer Center, New York, New York; 6 Department of Geriatrics, Memorial Sloan Kettering Cancer Center, New York, New York

## Abstract

**Importance:**

Many patients with metastatic cancer receive high-cost, low-value care near the end of life. Identifying patients with a high likelihood of receiving low-value care is an important step to improve appropriate end-of-life care.

**Objective:**

To analyze patterns of care and interventions during terminal hospitalizations and examine whether care management is associated with sociodemographic status among adult patients with metastatic cancer at the end of life.

**Design, Setting, and Participants:**

This retrospective, population-based cross-sectional study used data from the Healthcare Cost and Utilization Project to analyze all-payer, encounter-level information from multiple inpatient centers in the US. All utilization and hospital charge records from national inpatient sample data sets between January 1, 2010, and December 31, 2017 (n = 58 761 097), were screened. The final cohort included 21 335 patients 18 years and older at inpatient admission who had a principal diagnosis of metastatic cancer and died during hospitalization. Data for the current study were analyzed from January 1, 2010, to December 31, 2017.

**Exposures:**

Patient demographic characteristics, patient insurance status, hospital location, and hospital teaching status.

**Main Outcomes and Measures:**

Receipt of systemic therapy (including chemotherapy and immunotherapy), receipt of invasive mechanical ventilation, emergency department (ED) admission, time from hospital admission to death, and total charges during a terminal hospitalization.

**Results:**

Among 21 335 patients with metastatic cancer who had terminal hospitalizations between 2010 and 2017, the median age was 65 years (interquartile range, 56-75 years); 54.0% of patients were female; 0.5% were American Indian, 3.3% were Asian or Pacific Islander, 14.1% were Black, 7.5% were Hispanic, 65.9% were White, and 3.1% were identified as other; 58.2% were insured by Medicare or Medicaid, and 33.2% were privately insured. Overall, 63.2% of patients were admitted from the ED, 4.6% received systemic therapy, and 19.2% received invasive mechanical ventilation during hospitalization. Racial and ethnic minority patients had a higher likelihood of being admitted from the ED (Asian or Pacific Islander patients: odds ratio [OR], 1.43 [95% CI, 1.20-1.72]; *P* < .001; Black patients: OR, 1.39 [95% CI, 1.27-1.52]; *P* < .001; and Hispanic patients: OR, 1.45 [95% CI, 1.28-1.64]; *P* < .001), receiving invasive mechanical ventilation (Black patients: OR, 1.59 [95% CI, 1.44-1.75]; *P* < .001), and incurring higher total charges (Asian or Pacific Islander patients: OR, 1.35 [95% CI, 1.13-1.60]; *P* = .001; Black patients: OR, 1.23 [95% CI, 1.13-1.34]; *P* < .001; and Hispanic patients: OR, 1.50 [95% CI, 1.34-1.69]; *P* < .001) compared with White patients. Privately insured patients had a lower likelihood of being admitted from the ED (OR, 0.47 [95% CI, 0.44-0.51]; *P* < .001), receiving invasive mechanical ventilation (OR, 0.75 [95% CI, 0.69-0.82]; *P* < .001), and incurring higher total charges (OR, 0.64 [95% CI, 0.59-0.68]; *P* < .001) compared with Medicare and Medicaid beneficiaries.

**Conclusions and Relevance:**

In this study, patients with metastatic cancer from racial and ethnic minority groups and those with Medicare or Medicaid coverage were more likely to receive low-value, aggressive interventions at the end of life. Further studies are needed to evaluate the underlying factors associated with disparities at the end of life to implement prospective interventions.

## Key Points

**Question:**

Is variation in care management during terminal hospitalization among adults with metastatic cancer associated with sociodemographic status?

**Findings:**

In this cross-sectional study of 21 335 patients with metastatic cancer who died in the hospital, racial and ethnic minority patients and those with Medicare or Medicaid coverage were more likely to receive low-value, high-cost aggressive medical interventions at the end of life.

**Meaning:**

This study’s findings suggest that identifying and understanding factors associated with the observed disparities will be helpful to inform communications with patients with metastatic cancer about end-of-life care.

## Teaser

This cross-sectional study uses data from the Healthcare Cost and Utilization Project national inpatient sample between 2010 and 2017 to examine care management patterns during terminal hospitalization among adult patients with metastatic cancer.

## Introduction

Minimizing the burdens encountered by patients with terminal cancer and their families is an important aspect of end-of-life medical care planning. Current guidelines define and discourage aggressive, invasive, and expensive medical services that represent low-value care at the end of life. The American Society of Clinical Oncology and the National Quality Forum define aggressive care as multiple hospital, intensive care unit, or emergency department (ED) admissions in the last 30 days of life, receipt of chemotherapy in the last 14 days of life, or enrollment in hospice 3 days or fewer before death.^1,2^ In addition to these measures, previous studies characterizing end-of-life care among patients with advanced cancer have included death in an acute care facility, receipt of invasive mechanical ventilation, and receipt of other specific life-extending intensive care therapies as signs of aggressive care.^3,4,5,6,7,8^

Despite best practice guidelines from the American Society of Clinical Oncology and the National Quality Forum that discourage low-value end-of-life care, many patients with metastatic cancer continue to receive aggressive medical interventions throughout their final weeks of life.^9^ Patients with advanced cancer often experience a substantial worsening in health during their final months, occasioning inpatient admission and intensive procedures that add to the substantial emotional and financial burden of care during the final stages of illness^4,10,11,12^; yet, this intensive inpatient care often does not achieve end-of-life goals for patients and their families or caregivers.^13,14^ Furthermore, previous studies have reported higher rates of invasive end-of-life interventions and higher costs incurred among racial or ethnic minority individuals and patients with low socioeconomic status.^5,8,15,16,17,18,19,20^ Higher rates of life-extending medical interventions have been found among Black patients, prolonged hospitalizations among Black and Asian patients, and a higher likelihood of dying in the hospital among Black, Asian, and Hispanic patients compared with White patients.^5,8,17,18,19^

To our knowledge, no previous study has specifically examined inpatient deaths among those with metastatic cancer on a population level in the US. The current study analyzed records of patients who died in the hospital and therefore received at least 1 measure of low-value end-of-life care according to previously cited signs of aggressive care.^3,4,5,6,7,8^ We aimed to assess additional measures of low-value care among this patient population in the context of terminal hospitalization; these measures included receipt of mechanical ventilation and systemic therapy, duration of hospitalization, total costs, and ED admission as a sign of unplanned care. An analysis of terminal inpatient encounters provides insight into the intensity of care received immediately before death and highlights opportunities for improved end-of-life care planning among patients with terminal cancer. We ultimately aimed to examine sociodemographic patterns in the use of interventions and the associated costs of inpatient care among this national cohort in the final days before an in-hospital death.

## Methods

### Data Source and Study Population

**Figure 1.**
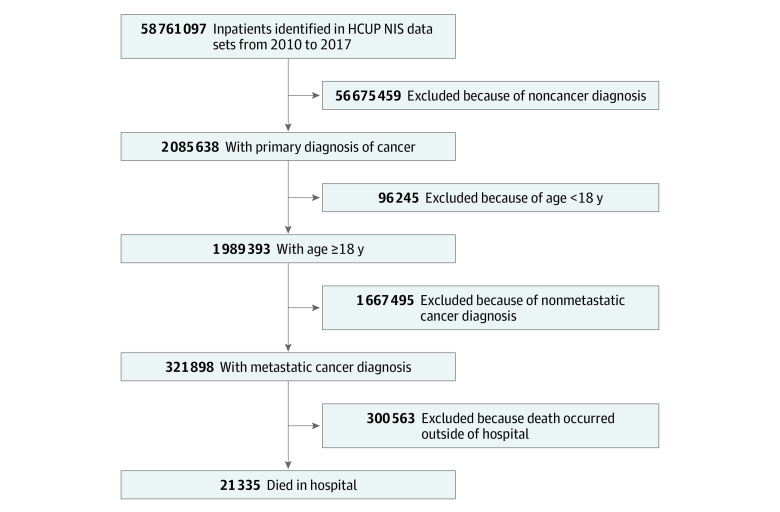
Study Flowchart HCUP indicates Healthcare Cost and Utilization Project; NIS, national inpatient sample.

This cross-sectional study analyzed data from the Agency for Healthcare Research and Quality Healthcare Cost and Utilization Project (HCUP) national inpatient sample, which includes 20% of all discharge records from community hospitals across the US.^21^ All records from national inpatient sample data sets between January 1, 2010, and December 31, 2017 (n = 58 761 097), were screened. Our cohort included all patients 18 years and older at hospital admission who had an in-hospital death and a principal diagnosis of metastatic cancer according to diagnostic codes from the *International Classification of Diseases, Ninth Revision, Clinical Modification* (*ICD-9-CM*); the *International Classification of Diseases, Tenth Revision, Clinical Modification* (*ICD-10-CM*); and the *International Classification of Diseases, Tenth Revision, Procedure Coding System* (*ICD-10-PCS*) (Figure 1). We selected records of patients with a principal diagnosis of secondary cancer to capture all hospitalizations associated with cancer that had spread from the primary site. Specific *ICD-9-CM* or *ICD-10-CM* diagnostic codes that were used to define the cohort are available in the eMethods and eTable 1 in the Supplement. We further selected records indicating death during hospitalization (defined as terminal hospitalization) to specifically examine inpatient services administered at the end of life. Institutional review board approval was waived by Memorial Sloan Kettering Cancer Center because all data used were public and deidentified. Data for the current study were analyzed from January 1, 2010, to December 31, 2017. The study followed the Strengthening the Reporting of Observational Studies in Epidemiology (STROBE) reporting guideline for cross-sectional studies.

### Exposures

Patient demographic characteristics included age, sex, race, ethnicity, socioeconomic status, and payer status. Race and ethnicity were derived from an HCUP-coded data element including the following categories: American Indian, Asian or Pacific Islander, Hispanic, non-Hispanic Black, non-Hispanic White, and other race or ethnicity (specific races and ethnicities included in this category were not available); HCUP coding includes race and ethnicity in 1 data element, with ethnicity taking precedence over race if the original record provided race and ethnicity separately.^22^ Socioeconomic status was obtained from the estimated median household income quartile of residents in the zip code on record, with quartile 1 representing the lowest income^22^ (eTable 2 in the Supplement). Payer status was derived from the primary expected payer on record, and categories were combined into 3 payer groups: (1) Medicare or Medicaid (public insurance), (2) private insurance, and (3) self-pay, no charges, or other insurance.^22^ Additional exposures included hospital census region, location, and teaching status.^22^

### Outcomes

We defined aggressive inpatient end-of-life services as the receipt of systemic therapy, including chemotherapy and immunotherapy, and/or the receipt of invasive mechanical ventilation during the terminal hospitalization, documented via HCUP clinical classification software, *ICD-9-CM* codes, and *ICD-10-PCS* codes (eTable 1 in the Supplement). We also included ED admission as a sign of unplanned care, which was coded if ED revenue codes, ED current procedural terminology, or positive ED charges were listed on the original discharge record. Additional outcomes included length of hospital stay as a measure of time from admission until death and total charges billed to insurance, not including professional fees and noncovered charges.^22^

### Statistical Analysis

We generated descriptive statistics using χ^2^ tests to assess univariate associations and Mann-Whitney U and Kruskal-Wallis tests to compare median values for continuous outcomes. We fit multivariable binomial logistic regression models to generate odds ratios (ORs) and 95% CIs, including all exposures as covariates for the following binary outcomes: ED admission, receipt of systemic therapy, receipt of mechanical ventilation, time from admission to death greater than the median value of the total cohort, and total charges billed to insurance greater than the median value of the total cohort. The preliminary analysis revealed no multicollinearity. We assessed and reported the inclusion of standardized residuals with a value greater than 2.5 SDs. Model significance and accuracy were evaluated using omnibus tests and analysis of receiver operating characteristic curves. We used and reported 2-tailed *P* values with a significance level of .05 for all comparisons. All statistical analyses were performed using SPSS software, version 27 (IBM SPSS Statistics).

## Results

Among 21 335 patients with metastatic cancer who had terminal hospitalizations between 2010 and 2017, the median age was 65 years (interquartile range [IQR], 56-75 years); 54.0% of patients were female, 45.9% were male; 0.5% were American Indian, 3.3% were Asian or Pacific Islander, 14.1% were Black, 7.5% were Hispanic, 65.9% were White, and 3.1% were identified as other (Table). Most patients (58.2%) had Medicare or Medicaid insurance, and 33.2% had private insurance; 63.2% of patients were admitted from the ED. A small proportion of patients (4.6%) received systemic therapy during terminal hospitalization, and a larger proportion (19.2%) received invasive mechanical ventilation. The median time from admission to death was 6 days (IQR, 3-12 days), and the median total charges were $43 681 (IQR, $17 973-$97 110). Complete descriptive and univariate data are shown in the Table as well as eTable 3 and eTable 4 in the Supplement.

**Table.** Cohort Sociodemographic Characteristics

Characteristic	No. (%)
Total participants, No.	21 335
Age, y	
Median (IQR) [range]^a^	65 (56-75) [18-100]
Group	
18-49	2710 (12.7)
50-59	4450 (20.9)
60-69	6103 (28.6)
≥70	8072 (37.8)
Sex	
Female	11 529 (54.0)
Male	9800 (45.9)
Missing	6 (0.03)
Race or ethnicity	
American Indian	101 (0.5)
Asian or Pacific Islander	696 (3.3)
Hispanic	1609 (7.5)
Non-Hispanic Black	3011 (14.1)
Non-Hispanic White	14 066 (65.9)
Other^b^	661 (3.1)
Missing	1191 (5.6)
Payer	
Medicare or Medicaid	12 424 (58.2)
Private insurance	7074 (33.2)
Other insurance	1770 (8.3)
Missing	67 (0.3)
Income quartile	
1 (Lowest income)	5555 (26.0)
2	4963 (23.3)
3	5015 (23.5)
4 (Highest income)	5281 (24.8)
Missing	521 (2.4)
Hospital location and teaching status	
Urban teaching	13 738 (64.4)
Urban nonteaching	5757 (27.0)
Rural	1763 (8.3)
Missing	77 (0.4)
Hospital region	
South	7750 (36.3)
Northeast	5479 (25.7)
West	4057 (19.0)
Midwest	4049 (19.0)
Year	
2010	3371 (15.8)
2011	3078 (14.4)
2012	2781 (13.0)
2013	2524 (11.8)
2014	2485 (11.6)
2015	2439 (11.4)
2016	2257 (10.6)
2017	2400 (11.2)
Admission from ED	13 489 (63.2)
Receipt of systemic therapy	986 (4.6)
Receipt of invasive mechanical ventilation	4087 (19.2)
Procedures, median (IQR) [range], No.^a^	2 (0-4) [0-30]
Time from admission to death, median (IQR) [range], d^c^	6 (3-12) [0-254]
Total charges billed to insurance, median (IQR) [range], $^d^	43 681 (17 973-97 110) [219-4 545 159]

Abbreviations: ED, emergency department; IQR, interquartile range.

^a^ Based on 21 335 participants.

^b^ Specific races and ethnicities included in this category were not available.

^c^ Based on 21 334 participants.

^d^ Based on 20 936 participants.

**Figure 2.**
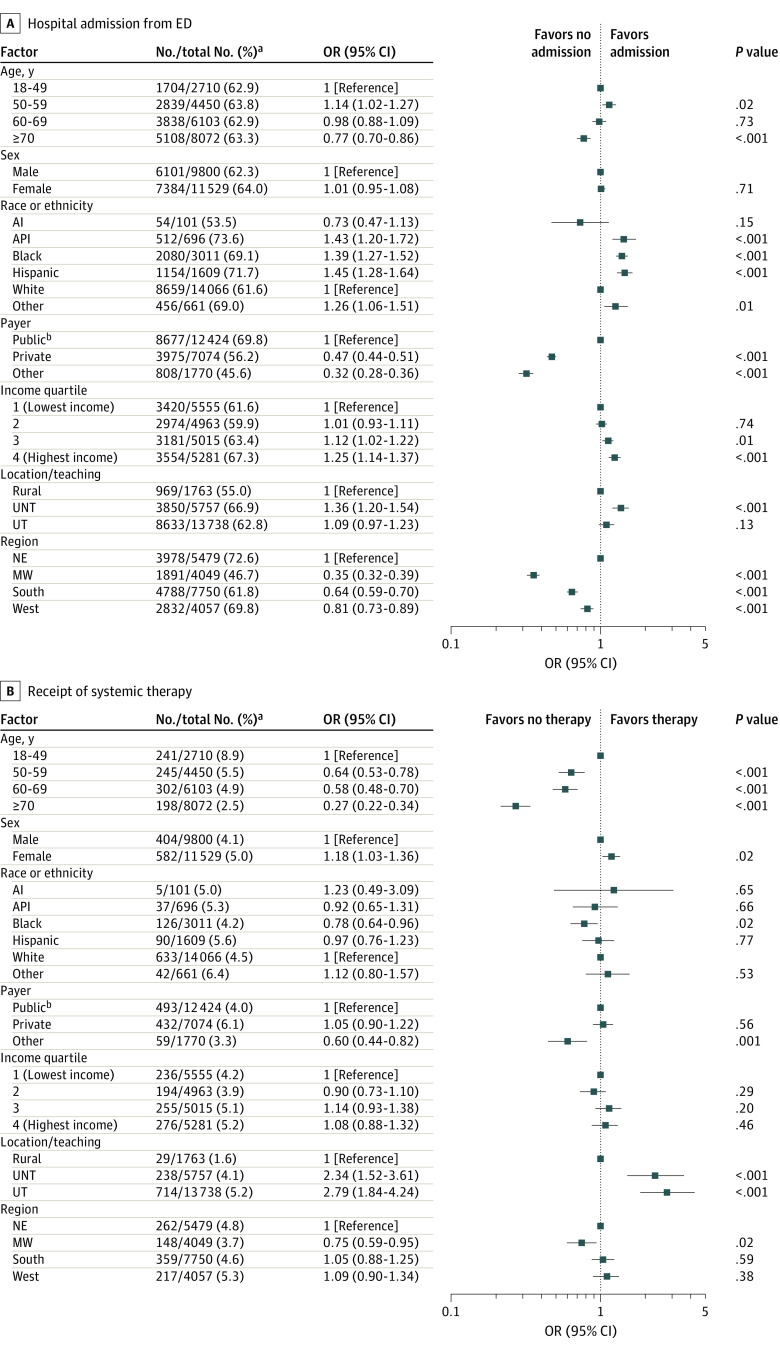
Association of Sociodemographic and Hospital Characteristics With Hospital Admission from ED and Receipt of Systemic Therapy AI indicates American Indian; API, Asian or Pacific Islander; ED, emergency department; MW, Midwest; NE, Northeast; OR, odds ratio; UNT, urban nonteaching; and UT, urban teaching. Specific races and ethnicities included in the *other* category were not available. ^a^Total numbers for subgroups differ because missing values for cases were excluded from the analysis. ^b^Includes Medicare and Medicaid coverage.

**Figure 3.**
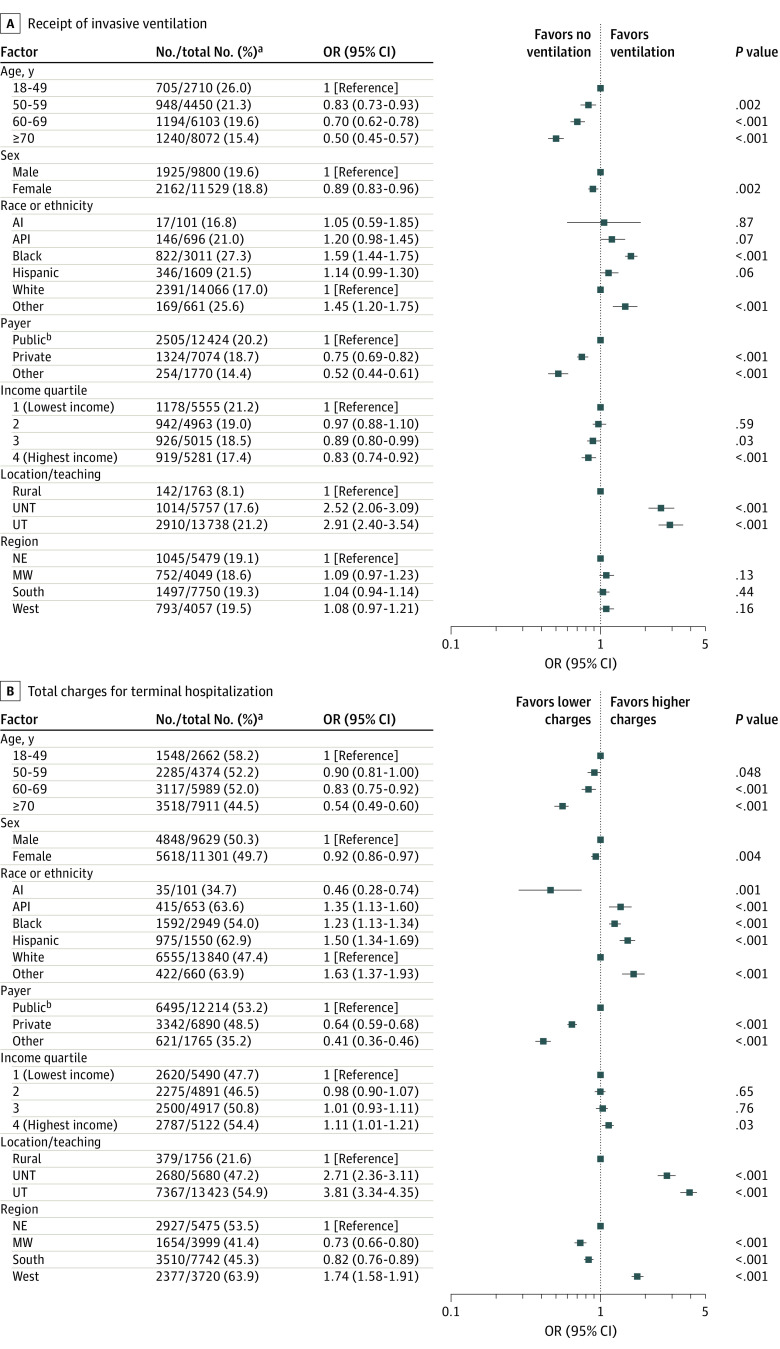
Association of Sociodemographic and Hospital Characteristics With Receipt of Invasive Ventilation and Total Charges B, Total charges (in US dollars) billed to insurance that were greater than the median value for the overall cohort. AI indicates American Indian; API, Asian or Pacific Islander; ED, emergency department; MW, Midwest; NE, Northeast; OR, odds ratio; UNT, urban nonteaching; and UT, urban teaching. Specific races and ethnicities included in the *other* category were not available.

In the multivariate analysis, several factors were associated with the receipt of aggressive inpatient services near the end of life. Overall, patients from several racial and ethnic minority groups, including Asian or Pacific Islander patients, Black patients, and Hispanic patients, and patients with Medicare or Medicaid coverage had a higher likelihood of being admitted from the ED, receiving more aggressive and higher-cost care, and having a longer terminal hospitalization compared with White patients and patients with private insurance. A higher likelihood of these outcomes was also observed among patients receiving care at urban hospitals, particularly urban teaching hospitals, compared with those receiving care at rural hospitals. Detailed results of the multivariable analysis are presented in the following paragraphs and shown in Figure 2, Figure 3, and eFigure 1 in the Supplement.

### ED Admission

A higher likelihood of ED admission was observed among patients aged 50 to 59 years (OR, 1.14; 95% CI, 1.02-1.27; *P* = .02); patients who were Asian or Pacific Islander (OR, 1.43; 95% CI, 1.20-1.72; *P* < .001), Black (OR, 1.39; 95% CI, 1.27-1.52; *P* < .001), and Hispanic (OR, 1.45; 95% CI, 1.28-1.64; *P* < .001); patients living in zip codes with higher median income quartiles (eg, quartile 4:, OR, 1.25; 95% CI, 1.14-1.37; *P* < .001); and patients receiving care at an urban nonteaching hospital (OR, 1.36; 95% CI, 1.20-1.54; *P* < .001) (Figure 2A). A lower likelihood of ED admission was observed among patients 70 years and older (OR, 0.77; 95% CI, 0.70-0.86; *P* < .001), patients with private insurance (OR, 0.47; 95% CI, 0.44-0.51; *P* < .001), self-payers or those with other insurance (OR, 0.32; 95% CI, 0.28-0.36; *P* < .001), and patients receiving care at a hospital located in the Midwest (OR, 0.35; 95% CI, 0.32-0.39; *P* < .001), South (OR, 0.64; 95% CI, 0.59-0.70; *P* < .001), or West (OR, 0.81; 95% CI, 0.73-0.89; *P* < .001).

### Systemic Therapy

A higher likelihood of receiving systemic therapy was observed among female patients (OR, 1.18; 95% CI, 1.03-1.36; *P* = .02) and patients receiving care at urban teaching (OR, 2.79; 95% CI, 1.84-4.24; *P* < .001) or urban nonteaching (OR, 2.34; 95% CI, 1.52-3.61; *P* < .001) hospitals (Figure 2B). A lower likelihood of receiving systemic therapy was observed among patients 70 years and older (OR, 0.27; 95% CI, 0.22-0.34; *P* < .001), Black patients (OR, 0.78; 95% CI, 0.64-0.96; *P* = .02), self-payers or those with other insurance (OR, 0.60; 95% CI, 0.44-0.82; *P* = .001), and patients receiving care at a hospital in the Midwest (OR, 0.75; 95% CI, 0.59-0.95; *P* = .02).

### Invasive Mechanical Ventilation

A higher likelihood of receiving invasive mechanical ventilation was observed among Black patients (OR, 1.59; 95% CI, 1.44-1.75; *P* < .001) and patients receiving care at urban teaching (OR, 2.91; 95% CI, 2.40-3.54; *P* < .001) or urban nonteaching (OR, 2.52; 95% CI, 2.06-3.09; *P* < .001) hospitals (Figure 3A). A lower likelihood of receiving invasive mechanical ventilation was observed among patients 70 years and older (OR, 0.50; 95% CI, 0.45-0.57; *P* < .001), female patients (OR, 0.89; 95% CI, 0.83-0.96; *P* = .002), patients with private insurance (OR, 0.75; 95% CI, 0.69-0.82; *P* < .001), self-payers or those with other insurance (OR, 0.52; 95% CI, 0.44-0.61; *P* < .001), and patients living in zip codes with higher median income quartiles (eg, quartile 4: OR, 0.83; 95% CI, 0.74-0.92; *P* < .001).

### Total Charges

A higher likelihood of greater total charges was observed among patients who were Asian or Pacific Islander (OR, 1.35; 95% CI, 1.13-1.60; *P* = .001), Black (OR, 1.23; 95% CI, 1.13-1.34; *P* < .001), Hispanic (OR, 1.50; 95% CI, 1.34-1.69; *P* < .001), and of other races and ethnicities (OR, 1.63; 95% CI, 1.37-1.93; *P* < .001); patients living in zip codes with higher median income quartiles (eg, quartile 4: OR, 1.11; 95% CI, 1.01-1.21; *P* = .03); patients receiving care at urban teaching (OR, 3.81; 95% CI, 3.34-4.35; *P* < .001) or urban nonteaching (OR, 2.71; 95% CI, 2.36-3.11; *P* < .001) hospitals; and patients receiving care at a hospital located in the West (OR, 1.74; 95% CI, 1.58-1.91; *P* < .001) (Figure 3B). A lower likelihood of greater total charges was observed among patients 70 years and older (OR, 0.54; 95% CI, 0.49-0.60; *P* < .001), female patients (OR, 0.92; 95% CI, 0.86-0.97; *P* = .004), American Indian patients (OR, 0.46; 95% CI, 0.28-0.74; *P* = .001), patients with private insurance (OR, 0.64; 95% CI, 0.59-0.68; *P* < .001), self-payers or those with other insurance (OR, 0.41; 95% CI, 0.36-0.46; *P* < .001), and patients receiving care at a hospital located in the Midwest (OR, 0.73; 95% CI, 0.66-0.80; *P* < .001) or South (OR, 0.82; 95% CI, 0.76-0.89; *P* < .001).

## Discussion

This cross-sectional study is the first, to our knowledge, to find sociodemographic disparities in the receipt of high-cost intensive services among patients with metastatic cancer during terminal hospitalization based on a contemporary, population-based cohort. These results highlight an unmet need for improved quality and equity of end-of-life care among patients with metastatic cancer who receive care management in the inpatient setting.

Previous studies have reported increased use of low-value end-of-life medical services among minority populations^5,8,17^; in particular, studies have found high rates of aggressive care among Black patients.^19,20^ Previous studies have also reported an association between greater use of aggressive medical interventions and lower rates of hospice enrollment among Asian, Black, and Hispanic patients with advanced cancer.^8,16,23,24^ Among our sample of patients with metastatic cancer who died in the hospital, minority race or ethnicity was associated with several measures of aggressive end-of-life care. Although previous work has found similar disparities,^5,8,17,19^ the current findings highlight existing disparities that are specifically associated with inpatient terminal cancer management. The current findings do not account for patterns of care in the home or outpatient setting, and we were not able to directly evaluate palliative care or hospice use in the study population. However, our observations in the context of previous findings suggest several aspects of patient care that may be associated with disparities in end-of-life inpatient care management: communication, cultural awareness, access to care, and structural racism.

The observed association between measures of high-cost, low-value care and minority race and ethnicity may reflect differences in cultural and environmental factors associated with preferences for end-of-life medical care as well as disparities in the use of palliative and advanced care planning services. Palliative care discussions and services enhance quality of life for both terminally ill patients and their caregivers and promote goal-concordant care, including the option of an at-home death.^3,14,25^ Palliative measures have also been associated with decreased use of aggressive, invasive, and expensive medical services^4,23,24,26,27^; thus, barriers to the use of palliative services may be associated with aggressive inpatient care at the end of life. Hispanic patients have cited limited information and education about advanced care planning from health care professionals as obstacles to creating an end-of-life care plan and receiving palliative care.^28,29^ In addition, among patients with limited English proficiency, professional interpreters are often used unsuccessfully or not at all, impairing patient and family understanding of illness, prognosis, and end-of-life medical decisions.^30^

Another factor that may be associated with end-of-life care disparities is variation in cultural awareness among health care professionals. Previous work has found a greater preference for life-extending treatments among Black patients, particularly among those who reported relying on spirituality to cope with terminal illness.^31,32,33,34^ Additional studies have highlighted faith-based objections to hospice enrollment among Hispanic patients.^23,24,28,34^ Comprehensive goals-of-care discussions require that the practitioner be able to recognize and address patient preferences that are based on personal and cultural beliefs. Cultural barriers between patient and practitioner may influence patient decisions to pursue aggressive therapies and perpetuate the use of invasive interventions among terminally ill ethnic and racial minority patients.^23,24,28,30,34^ In addition, physician factors, including practice norms and expectations regarding patient goals, have been cited as factors associated with challenges in successful communication regarding end-of-life care management.^35,36^ These obstacles highlight opportunities to promote high-value care for minority patients through improved patient-practitioner communication and understanding.

In addition to communication barriers, obstacles to accessing health care have been associated with lower rates of hospice enrollment among racial and ethnic minority individuals and may therefore be associated with greater use of aggressive care. The criteria for hospice enrollment may alienate racial and ethnic minority patients; these criteria are variable and can require a prognosis of 6 months or less from a primary care physician, the presence of physical symptoms, and an obligation to forgo curative treatments.^15,23,37^ Many ethnic and racial minority patients have lower incomes and limited insurance coverage, and those with undocumented immigration status do not qualify for Medicaid coverage in many states.^23,24^ The higher likelihood of ED admission among Asian, Black, and Hispanic patients in the current sample may suggest unplanned end-of-life care and underlying barriers to accessing advanced care planning services that may limit the use of aggressive inpatient care at the end of life.

The least studied and arguably the biggest challenge in addressing cancer care disparities is structural bias.^38,39,40^ Previous work has reported high rates of aggressive care and low rates of advanced care planning among minority groups despite previous end-of-life care discussions with a practitioner^24,41^ as well as fewer positive nonverbal communication cues from practitioners during end-of-life care discussions with Black patients compared with White patients.^42^ These findings necessitate further investigation of the challenges encountered by patients from minority groups regarding end-of-life care management that may be associated with other factors, including possible racial discrimination.

In addition, the current study found greater use of low-value end-of-life care among patients with Medicare or Medicaid coverage compared with private insurance; this finding is consistent with those of previous studies reporting low-value care among publicly insured patients^9,43^ and suggests a pattern in the context of inpatient care received during terminal hospitalization among a large national cohort of patients with metastatic cancer. Previous work has found high rates of intensive care, low rates of routine goals-of-care discussions, and low rates of hospice use among Medicare beneficiaries in the general population^23,43,44,45,46^; 1 study has also reported increasing rates of late hospice enrollment and late hospitalizations among this population in the past 13 years.^9^ A substantial proportion of Medicare spending is devoted to hospital inpatient care. In 2018, payments for inpatient care accounted for $137 billion of Medicare spending compared with $19.3 billion for hospice care.^47^ Similar patterns have been found among Medicaid beneficiaries, albeit with even lower hospice use.^17,24,48^ Further examination of barriers to palliative care use among publicly insured patients is warranted to understand factors associated with the greater likelihood of aggressive care observed among this group.

Although we define markers of low-value end-of-life care, the value of care is ultimately determined by a patient’s goals. Depending on a particular patient’s end-of-life wishes, goal-concordant care can encompass interventions that aim to maximize patient comfort as well as intensive life-extending interventions. In addition, although ED admission may be a sign of unplanned care for some patients, others may be transferred from a clinic to the ED for direct admission as part of routine care. Accurately defining goal-concordant care is dependent on context, and individual considerations must be balanced when making end-of-life care decisions.^49,50^

The possible factors associated with disparities in end-of-life care among patients with metastatic cancer are complex, encompassing patient-practitioner communication, cultural preferences, access to care, and other systemic factors, including biases. Investigating and identifying specific barriers to high-value care encountered by racial and ethnic minority patients and patients with public insurance will be important in designing targeted interventions.^51,52,53^

### Limitations

This study has limitations. The study is retrospective and observational; therefore, the associations reported cannot be equated with causation. Our multivariate model does not adjust for unmeasured confounders or completely eliminate sociodemographic confounding factors. The cross-sectional study design reflects a single inpatient stay and does not reflect level of care, health care use, and costs before terminal hospitalization; the current analysis also pertains to data specific to the inpatient setting only and does not examine end-of-life care administered in the home or other outpatient settings. In addition, specific practice patterns may be associated with individual hospitals, and patients receiving treatment at the same facilities may experience similar care management approaches compared with patients with equivalent circumstances receiving treatment at different facilities; however, we were unable to account for within-center differences in the analysis.

The immediate cause of death among patients in the sample is unknown; a proportion of in-hospital deaths analyzed may not be directly associated with terminal cancer and may instead be associated with unexpected acute illness or treatment complications. Metastatic cancer diagnoses are established through the primary diagnosis, which is defined as the condition associated with inpatient admission, and do not represent cancer registry diagnoses with more specific prognostic information. In addition, it is possible that patients with metastatic cancer may have been admitted using the diagnostic code corresponding to the primary cancer site and thus were not captured in this study. Further data regarding end-of-life care, including hospice enrollment, previous goals-of-care conversations, and comorbid conditions, are not available in the data but can have substantial implications for the course of treatment.

## Conclusions

Among patients with metastatic cancer who died in the hospital, increased rates of aggressive high-cost care were associated with Black and Asian or Pacific Islander race, Hispanic ethnicity, public insurance status, and admission to an urban teaching hospital. This study identified groups at risk of receiving high-cost, low-value interventions that may oppose the patient’s goals and exacerbate the physical, emotional, and financial burdens of terminal cancer. Future directions include qualitative studies assessing patient perspectives on end-of-life discussions and care options, including palliative services. Interventions may focus on initiating discussions and palliative measures earlier as well as facilitating discussions that emphasize assessing patient goals and optimizing patient education about care options. Identifying these disparities is important in guiding interventions designed to improve accessibility and the equitable use of high-value end-of-life services for patients with metastatic cancer.

## Notes

Supplement. **eMethods.** Diagnosis and Procedures, Exposures, Outcomes, and Statistical Analysis **eTable 1.***ICD* Diagnosis and Procedure Codes Used to Define the Cohort and Outcomes **eTable 2.** Yearly Quartile Ranges of Median Household Income According to Zip Code **eTable 3.** Chi-squared Comparisons of Outcomes by Demographic Characteristics **eTable 4.** Mann-Whitney U Test Used for Comparison of Medians by Sex and Kruskal-Wallis Test Used for All Other Comparisons **eFigure.** Median Time From Hospital Admission Until Death Greater Than Median for Overall Cohort
eReferences
