# Supplementary material for: Disparities in Care Management During Terminal Hospitalization Among Adults With Metastatic Cancer From 2010 to 2017
Source: JAMA Netw Open. 2021 Sep 22;4(9):e2125328. doi: 10.1001/jamanetworkopen.2021.25328 (PMC8459194; doi:10.1001/jamanetworkopen.2021.25328)
Supplement: Supplement. — eMethods. Diagnosis and Procedures, Exposures, Outcomes, and Statistical Analysis eTable 1.ICD Diagnosis and Procedure Codes Used to Define the Cohort and Outcomes eTable 2. Yearly Quartile Ranges of Median Household Income According to Zip Code eTable 3. Chi-squared Comparisons of Outcomes by Demographic Characteristics eTable 4. Mann-Whitney U Test Used for Comparison of Medians by Sex and Kruskal-Wallis Test Used for All Other Comparisons eFigure. Median Time From Hospital Admission Until Death Greater Than Median for Overall Cohort eReferences [file jamanetwopen-e2125328-s001.pdf]

Supplementary Online Content

Deeb S, Chino FL, Diamond LC, et al. Disparities in care management during terminal hospitalization among adults with metastatic cancer from 2010 to 2017. *JAMA Netw Open*. 2021;4(9):e2125328. doi:10.1001/jamanetworkopen.2021.25328

eMethods. Diagnosis and Procedures, Exposures, Outcomes, and Statistical Analysis

eTable 1. *ICD* Diagnosis and Procedure Codes Used to Define the Cohort and Outcomes

eTable 2. Yearly Quartile Ranges of Median Household Income According to Zip Code

eTable 3. Chi-squared Comparisons of Outcomes by Demographic Characteristics

eTable 4. Mann-Whitney U Test Used for Comparison of Medians by Sex and Kruskal-Wallis Test Used for All Other Comparisons

eFigure. Median Time From Hospital Admission Until Death Greater Than Median for Overall Cohort

eReferences

This supplementary material has been provided by the authors to give readers additional information about their work.

© 2021 Deeb S et al. *JAMA Network Open*.

eMethods. Diagnosis and Procedures, Exposures, Outcomes, and Statistical Analysis

Diagnosis and procedures

The NIS includes the International Classification of Diseases, Ninth Revision, Clinical Modification (ICD-9-CM) diagnosis and procedure codes prior to October 1, 2015 and International Classification of Diseases, Tenth Revision, Clinical Modification/Procedure Coding System (ICD-9-CM/PCS) diagnosis and procedure codes beginning October 1, 2015[1]. The US transitioned to the ICD-10-CM/PCS coding scheme on October 1, 2015, at the juncture of the third and fourth discharge quarters reported in each NIS patient record; our analysis includes records containing ICD-9-CM codes as well as records containing ICD-10-CM/PCS codes. As recommended by HCUP guidelines, we analyzed data from 2015, the year of this transition, by discharge quarter in order to assess changes in reported frequencies of diagnosis and procedure codes of interest due to the change in classification scheme. We found the reporting of metastatic cancer diagnoses and procedures of interest, according to the ICD codes listed in eTable 1, to be continuous across this transition. We therefore report compiled data spanning years 2010-2017. The NIS includes codes for a variable number of procedures depending on the state of origin of the record; we analyzed the first 15 procedures, which was the maximum number of reported procedures through 2015[2].

Exposures

Socioeconomic status

The HCUP reports the estimated median household income of residents in a patient's ZIP code, based on values derived from ZIP code-demographic data obtained from Claritas. The HCUP uses a categorical quartile classification, with the fourth quartile representing the wealthiest population. These estimates update annually and the dollar value ranges represented by each quartile vary between the 2010-2017 span of our dataset; this is reported in eTable 2[2].

Payer status

The HCUP coding combines detailed payer categories into more broad groups to allow uniformity of coding across records from different states. "Medicare" as primary expected payer includes both fee-for-service and managed care Medicare patients. "Medicaid" includes both fee-for-service and managed care Medicaid patients. "Private insurance" includes Blue Cross, commercial carriers, and private HMOs and PPOs. "Other" includes Worker's Compensation, CHAMPUS, CHAMPVA, Title V, and other government programs[2]. For this study, these categories were further combined into three payer groups: (1) Medicare or Medicaid, (2) private insurance, or (3) self-pay, no charges, or other.

© 2021 Deeb S et al. *JAMA Network Open*.

Hospital characteristics

The HCUP obtained hospital location and teaching status from the AHA Annual Survey of Hospitals, and defined “urban” hospitals as being located in a metropolitan statistical area and “rural” hospitals as being located in a non-metropolitan statistical area. Urban hospitals were subdivided according to teaching status, with “urban teaching” hospitals defined as either having an AMA-approved residency program, being a member of the Council of Teaching Hospitals (COTH), or having a ratio of full-time equivalent interns and residents to beds of .25 or higher. Rural hospitals were not subdivided by teaching status because this category was rare. The HCUP also obtained hospital census region from the AHA Annual Survey of Hospitals, with census region defined by the U.S. Census Bureau as either Northeast, Midwest, South, or West[2].

Outcomes

Admission through the ED

The HCUP defines a data element indicating records with evidence of emergency department (ED) services, and guidelines indicate this data element as the most comprehensive indicator available in the NIS of inpatient admissions through the ED. Services captured by this data element include ED revenue codes of 450-459 on record, a positive ED charge (when revenue center codes are not available), ED CPT codes (99281-99285) reported on record, a condition code of P7 reported on record, point of origin of ED, or admission source of ED[2]. Alternative non-ED admission sources include transfers from a different acute care hospital, transfers from a different health facility including long-term care facilities, admission from court or law enforcement, admission from an outpatient facility or clinic, or physician referral[2]. The HCUP acknowledges the possibility of a patient incurring ED services with coding information that may not have been captured in the HCUP record, and therefore the possible under-representation of ED admissions based on HCUP coding. We utilized this data element as an indicator of patient admission from the ED.

Time from hospital admission to death

The HCUP codes the length of the inpatient stay in days, calculated by subtracting the admission date from the discharge date, ranging from 0 (same-day stay) to 365 days, and without subtracting leave days. The HCUP utilizes the length of stay supplied from the data source if it cannot be calculated, and codes it as missing if it cannot be calculated and is not supplied[2].

Total charges billed to insurance

© 2021 Deeb S et al. *JAMA Network Open*.

The HCUP reports total charges billed to insurance, generally not including professional fees and non-covered charges. These NIS reported total charges may include emergency department charges incurred prior to hospital admission, as Medicare requires a bundled bill for patients admitted to the hospital through the emergency department. HCUP coding rounds charges to the nearest dollar and sets zero charges as missing; HCUP coding of total charges was analyzed in this study[2].

Systemic therapy

Patients were coded as receiving systemic therapy during their inpatient stay if one of the first 15 coded HCUP procedure CCS codes was 224 (“cancer chemotherapy”), a category which includes both systemic chemotherapy and immunotherapy procedures. The ICD-9-CM and ICD-10-CM/PCS codes corresponding to specific procedures within this category are listed in eTable 1.

Invasive mechanical ventilation

Patients were coded as receiving invasive mechanical ventilation during their inpatient stay if one of the first 15 coded ICD-9-CM and ICD-10-CM/PCS procedures was one of the codes corresponding to this intervention, listed in eTable 1. These codes were selected based on their inclusion in the HCUP procedure CCS category 216 (“respiratory intubation and mechanical ventilation”), with specific ICD codes indicating non-invasive mechanical ventilation removed.

Statistical analysis

(Multicollinearity analysis was run prior to performing the multivariable binomial logistic regressions, using dummy variables representing each covariate and linear regression. For each model, all VIF values were less than 5 indicating no multicollinearity. Additionally, standardized residuals with a value greater than 2.5 standard deviations were assessed and reported. Significance was assessed according to Omnibus tests for each model, and accuracy was evaluated by generating ROC curves to obtain AUC values. The SPSS syntax utilized in this analysis is available upon request.

© 2021 Deeb S et al. *JAMA Network Open*.

eTable 1. *ICD* Diagnosis and Procedure Codes Used to Define the Cohort and Outcomes

Diagnosis/ Intervention	Source	System	Codes included
Metastatic cancer	Primary diagnosis	ICD-9 diagnosis codes	'1960' '1961' '1962' '1963' '1965' '1966' '1968' '1969' '1970' '1971' '1972' '1973' '1974' '1975' '1976' '1977' '1978' '1980' '1981' '1982' '1983' '1984' '1985' '1986' '1987' '19881' '19882' '19889' '20971' '20972' '20973' '20974' '51181' '78951' '1990' '20970' '20975' '20979'
		ICD-10 diagnosis codes	'C770' 'C771' 'C772' 'C773' 'C774' 'C775' 'C778' 'C779' 'C7800' 'C7801' 'C7802' 'C781' 'C782' 'C7830' 'C7839' 'C784' 'C785' 'C786' 'C787' 'C7880' 'C7889' 'C7900' 'C7901' 'C7902' 'C7910' 'C7911' 'C7919' 'C792' 'C7931' 'C7932' 'C7940' 'C7949' 'C7951' 'C7952' 'C7960' 'C7961' 'C7962' 'C7970' 'C7971' 'C7972' 'C7981' 'C7982' 'C7989' 'C799' 'C800' 'C7B00' 'C7B01' 'C7B02' 'C7B03' 'C7B04' 'C7B09' 'C7B1' 'C7B8'
Admission from ED	HCUP ED admission data element		1: 'ED revenue code on record'<br>2: 'Positive ED charge (when revenue center codes not available)'<br>3: 'ED CPT procedure code on record'<br>4: 'Condition code P7, indication of ED admission, point of origin of ED, or admission source of ED'
Invasive Mechanical Ventilation	Procedures 1-15	ICD-9 procedure codes	'9392' '9601' '9602' '9603' '9604' '9605' '9670' '9671' '9672'
		ICD-10 procedure codes	'09HN7BZ' '09HN8BZ' '0BH13EZ' '0BH17EZ' '0BH18EZ' '0CHY7BZ' '0CHY8BZ' '0DH57BZ' '0DH58BZ' '0WHQ73Z' '0WHQ7YZ' '5A1935Z' '5A1945Z' '5A1955Z'
Systemic Therapy	Procedures 1-15	HCUP CCS category	'224'
		ICD-9 procedure codes	'0010' '0015' '1770' '9925' '9928'
		ICD-10 procedure codes	'3E00X05' '3E00X0M' '3E01305' '3E0130M' '3E02305' '3E0230M' '3E03002' '3E03003' '3E03005' '3E0300M' '3E0300P' '3E030GN' '3E03302' '3E03303' '3E03305' '3E0330M' '3E0330P' '3E033GN' '3E04002' '3E04003' '3E04005' '3E0400M' '3E0400P' '3E040GN' '3E04302' '3E04303' '3E04305' '3E0430M' '3E0430P' '3E043GN' '3E05002' '3E05003' '3E05005' '3E0500M' '3E0500P' '3E050GN' '3E05302' '3E05303' '3E05305' '3E0530M' '3E0530P' '3E053GN' '3E06002' '3E06003' '3E06005' '3E0600M' '3E0600P' '3E060GN' '3E06302' '3E06303' '3E06305' '3E0630M' '3E0630P' '3E063GN' '3E09305' '3E0930M' '3E09705' '3E0970M' '3E09X05' '3E09X0M' '3E0A305' '3E0A30M' '3E0B305' '3E0B30M' '3E0B705' '3E0B70M' '3E0BX05' '3E0BX0M' '3E0C305' '3E0C30M' '3E0C705' '3E0C70M' '3E0CX05' '3E0CX0M' '3E0D305' '3E0D30M' '3E0D705' '3E0D70M' '3E0DX05' '3E0DX0M' '3E0E305' '3E0E30M' '3E0E705' '3E0E70M' '3E0E805' '3E0E80M' '3E0F305' '3E0F30M' '3E0F705' '3E0F70M'

© 2021 Deeb S et al. *JAMA Network Open*.

			'3E0F805' '3E0F80M' '3E0G305' '3E0G30M' '3E0G705' '3E0G70M' '3E0G805' '3E0G80M' '3E0H305' '3E0H30M' '3E0H705' '3E0H70M' '3E0H805' '3E0H80M' '3E0J305' '3E0J30M' '3E0J705' '3E0J70M' '3E0J805' '3E0J80M' '3E0K305' '3E0K30M' '3E0K705' '3E0K70M' '3E0K805' '3E0K80M' '3E0L305' '3E0L30M' '3E0L705' '3E0L70M' '3E0M305' '3E0M30M' '3E0M30Y' '3E0M705' '3E0M70M' '3E0N305' '3E0N30M' '3E0N705' '3E0N70M' '3E0N805' '3E0N80M' '3E0P305' '3E0P30M' '3E0P705' '3E0P70M' '3E0P805' '3E0P80M' '3E0Q005' '3E0Q00M' '3E0Q305' '3E0Q30M' '3E0Q705' '3E0Q70M' '3E0R302' '3E0R303' '3E0R305' '3E0R30M' '3E0S302' '3E0S303' '3E0S305' '3E0S30M' '3E0U305' '3E0U30M' '3E0V305' '3E0V30M' '3E0W305' '3E0W30M' '3E0Y305' '3E0Y30M' '3E0Y705' '3E0Y70M' 'XW03351' 'XW033B3' 'XW033C3' 'XW033Q5' 'XW033S5' 'XW04351' 'XW043B3' 'XW043C3' 'XW043Q5' 'XW043S5' 'XW0DXJ5' 'XW0DXL5' 'XW0DXR5' 'XW0DXV5'

© 2021 Deeb S et al. *JAMA Network Open*.

eTable 2. Yearly Quartile Ranges ($) of Median Household Income According to Zip Code

Year	Quartile 1	Quartile 2	Quartile 3	Quartile 4
**2010**	1 - 40,999	41,000 - 50,999	51,000 - 66,999	67,000+
**2011**	1 - 38,999	39,000 - 47,999	48,000 - 63,999	64,000+
**2012**	1 - 38,999	39,000 - 47,999	48,000 - 62,999	63,000+
**2013**	1 - 37,999	38,000 - 47,999	48,000 - 63,999	64,000+
**2014**	1 - 39,999	40,000 - 50,999	51,000 - 65,999	66,000+
**2015**	1 - 41,999	42,000 - 51,999	52,000 - 67,999	68,000+
**2016**	1 - 42,999	43,000 - 53,999	54,000 - 70,999	71,000+
**2017**	1 - 43,999	44,000 - 55,999	56,000 - 73,999	74,000+

© 2021 Deeb S et al. *JAMA Network Open*.

eTable 3. Chi-squared Comparisons of Outcomes by Demographic Characteristics

Characteristics[a]		Systemic therapy (N/N_{tot} (%))[b]	p value	Invasive ventilation (N/N_{tot} (%))[b]	p value	Admission from ED (N/N_{tot} (%))[b]	p value
**Age (y)**	18-49	241/2710 (8.9)	<0.0001	705/2710 (26.0)	<0.0001	1704/2710 (62.9)	0.78
	50-59	245/4450 (5.5)		948/4450 (21.3)		2839/4450 (63.8)	
	60-69	302/6103 (4.9)		1194/6103 (19.6)		3838/6103 (62.9)	
	≥70	198/8072 (2.5)		1240/8072 (15.4)		5108/8072 (63.3)	
**Sex**	Male	404/9800 (4.1)	0.0015	1925/9800 (19.6)	0.10	6101/9800 (62.3)	0.0071
	Female	582/11529 (5.0)		2162/11529 (18.8)		7384/11529 (64.0)	
**Race/ ethnicity**	White	633/14066 (4.5)	0.063	2391/14066 (17.0)	<0.0001	8659/14066 (61.6)	<0.0001
	Black	126/3011 (4.2)		822/3011 (27.3)		2080/3011 (69.1)	
	Hispanic	90/1609 (5.6)		346/1609 (21.5)		1154/1609 (71.7)	
	API	37/696 (5.3)		146/696 (21.0)		512/696 (73.6)	
	NA	5/101 (5.0)		17/101 (16.8)		54/101 (53.5)	
	Other	42/661 (6.4)		169/661 (25.6)		456/661 (69.0)	
**Payer**	Public	493/12424 (4.0)	<0.0001	2505/12424 (20.2)	<0.0001	8677/12424 (69.8)	<0.0001
	Private	432/7074 (6.1)		1324/7074 (18.7)		3975/7074 (56.2)	
	Other	59/1770 (3.3)		254/1770 (14.4)		808/1770 (45.6)	
**Income quartile**	First	236/5555 (4.2)	0.0025	1178/5555 (21.2)	<0.0001	3420/5555 (61.6)	<0.0001
	Second	194/4963 (3.9)		942/4963 (19.0)		2974/4963 (59.9)	
	Third	255/5015 (5.1)		926/5015 (18.5)		3181/5015 (63.4)	
	Fourth	276/5281 (5.2)		919/5281 (17.4)		3554/5281 (67.3)	
**Location/ teaching**	Rural	29/1763 (1.6)	<0.0001	142/1763 (8.1)	<0.0001	969/1763 (55.0)	<0.0001
	UNT	238/5757 (4.1)		1014/5757 (17.6)		3850/5757 (66.9)	
	UT	714/13738 (5.2)		2910/13738 (21.2)		8633/13738 (62.8)	
**Region**	NE	262/5479 (4.8)	0.0032	1045/5479 (19.1)	0.696	3978/5479 (72.6)	<0.0001
	MW	148/4049 (3.7)		752/4049 (18.6)		1891/4049 (46.7)	
	South	359/7750 (4.6)		1497/7750 (19.3)		4788/7750 (61.8)	
	West	217/4057 (5.3)		793/4057 (19.5)		2832/4057 (69.8)	

[a] Abbreviations: White = White, non-Hispanic; Black = Black, non-Hispanic API = Asian/Pacific Islander; NA = Native American; public = Medicare or Medicaid; UNT = urban non-teaching; UT = urban teaching; NE = Northeast; MW = Midwest.
[b] N_{tot} for subgroups differ as missing values were excluded case-wise from analysis.

© 2021 Deeb S et al. *JAMA Network Open*.

eTable 4. Mann-Whitney U Test Used for Comparison of Medians by Sex and Kruskal-Wallis Test Used for All Other Comparisons

**Characteristics**[a]		**N**[b]	**Median (Q1, Q3) time until death (days)**	**p value**	**N**[b]	**Median (Q1, Q3) total charges ($)**	**p value**
**Age (y)**	18-49	2710	7 (3, 14)	<0.0001	2662	57003.50 (24664.25, 129909.00)	<0.0001
	50-59	4449	6 (3, 12)		4374	47259.00 (18864.75, 106084.00)	
	60-69	6103	7 (3, 12)		5989	46230.00 (18976.50, 99161.00)	
	≥70	8072	6 (3, 11)		7911	37174.00 (15339.00, 81681.00)	
**Sex**	Male	9800	6 (3, 12)	0.13	9629	44256.00 (17988.50, 100878.50)	0.10
	Female	11528	6 (3, 12)		11301	43352.00 (17950.50, 94474.00)	
**Race/ ethnicity**	White	14065	6 (3, 11)	<0.0001	13840	40032.00 (16579.50, 89859.00)	<0.0001
	Black	3011	7 (3, 14)		2949	49184.00 (20593.50, 108463.00)	
	Hispanic	1609	7 (3, 14)		1550	63313.00 (27446.00, 135367.75)	
	API	696	7 (3, 13)		653	67812.00 (28576.50, 135849.50)	
	NA	101	5 (2, 9)		101	33374.00 (11554.00, 75928.50)	
	Other	661	8 (4, 14)		660	68016.50 (29067.00, 140791.25)	
**Payer**	Public	12423	7 (3, 12)	<0.0001	12214	48056.50 (22315.50, 100691.25)	<0.0001
	Private	7074	6 (3, 11)		6890	41287.50 (15179.50, 97783.25)	
	Other	1770	5 (2, 10)		1765	22862.00 (4990.50, 71455.00)	
**Income quartile**	First	5554	6 (3, 12)	<0.0001	5490	41035.00 (17180.25, 91646.50)	<0.0001
	Second	4963	6 (3, 11)		4891	39112.00 (15953.00, 85570.00)	
	Third	5015	6 (3, 12)		4917	44655.00 (18998.50, 101563.50)	
	Fourth	5281	7 (3, 12)		5122	50785.50 (20093.75, 110014.50)	
**Location/ teaching**	Rural	1763	5 (2, 8)	<0.0001	1756	17527.50 (8115.00, 38271.00)	<0.0001
	UNT	5757	6 (3, 11)		5680	40229.50 (15906.50, 89772.25)	
	UT	13737	7 (3, 13)		13423	50754.00 (21790.00, 109227.00)	
**Region**	NE	5479	7 (3, 13)	<0.0001	5475	48739.00 (20722.00, 109539.00)	<0.0001
	MW	4049	6 (3, 10)		3999	33742.00 (14228.00, 71620.00)	
	South	7749	6 (3, 12)		7742	37754.50 (14815.25, 83514.50)	
	West	4057	6 (3, 12)		3720	66825.00 (28995.50, 144213.25)	

[a] Abbreviations: White = White, non-Hispanic; Black = Black, non-Hispanic API = Asian/Pacific Islander; NA = Native American; public = Medicare or Medicaid; UNT = urban non-teaching; UT = urban teaching; NE = Northeast; MW = Midwest.
[b] N for subgroups differ as missing values were excluded case-wise from analysis.

© 2021 Deeb S et al. *JAMA Network Open*.

eFigure. Median Time (Days) From Hospital Admission Until Death Greater Than Median for Overall Cohort

Factors[a]		N/N_{tot}(%)[b]	OR (95% CI)	p value
Age (y)	18-49	1399/2710 (51.6)	1	
	50-59	2204/4449 (49.5)	0.98 (0.89-1.09)	0.74
	60-69	3098/6103 (50.8)	0.98 (0.89-1.08)	0.68
	≥70	3701/8072 (45.8)	0.75 (0.68-0.83)	<0.0001
Sex	Male	4725/9800 (48.2)	1	
	Female	5674/11528 (49.2)	1.01 (0.96-1.07)	0.62
Race/ ethnicity	White	6619/14065 (47.1)	1	
	Black	1622/3011 (53.9)	1.21 (1.11-1.32)	<0.0001
	Hispanic	826/1609 (51.3)	1.10 (0.99-1.23)	0.078
	API	371/696 (53.3)	1.21 (1.03-1.41)	0.021
	NA	40/101 (39.6)	0.73 (0.47-1.14)	0.16
	Other	392/661 (59.3)	1.48 (1.25-1.75)	<0.0001
Payer	Public	6399/12423 (51.5)	1	
	Private	3310/7074 (46.8)	0.72 (0.67-0.77)	<0.0001
	Other	674/1770 (38.1)	0.54 (0.48-0.60)	<0.0001
Income quartile	First	2698/5554 (48.6)	1	
	Second	2296/4963 (46.3)	0.97 (0.89-1.05)	0.41
	Third	2463/5015 (49.1)	1.04 (0.96-1.13)	0.34
	Fourth	2659/5281 (50.4)	1.06 (0.98-1.16)	0.16
Location/ teaching	Rural	641/1763 (36.4)	1	
	UNT	2531/5757 (44.0)	1.29 (1.15-1.46)	<0.0001
	UT	7191/13737 (52.3)	1.72 (1.54-1.93)	<0.0001
Region	NE	2935/5479 (53.6)	1	
	MW	1789/4049 (44.2)	0.77 (0.70-0.84)	<0.0001
	South	3732/7749 (48.2)	0.87 (0.81-0.94)	0.00032
	West	1946/4057 (48.0)	0.85 (0.78-0.93)	0.00025

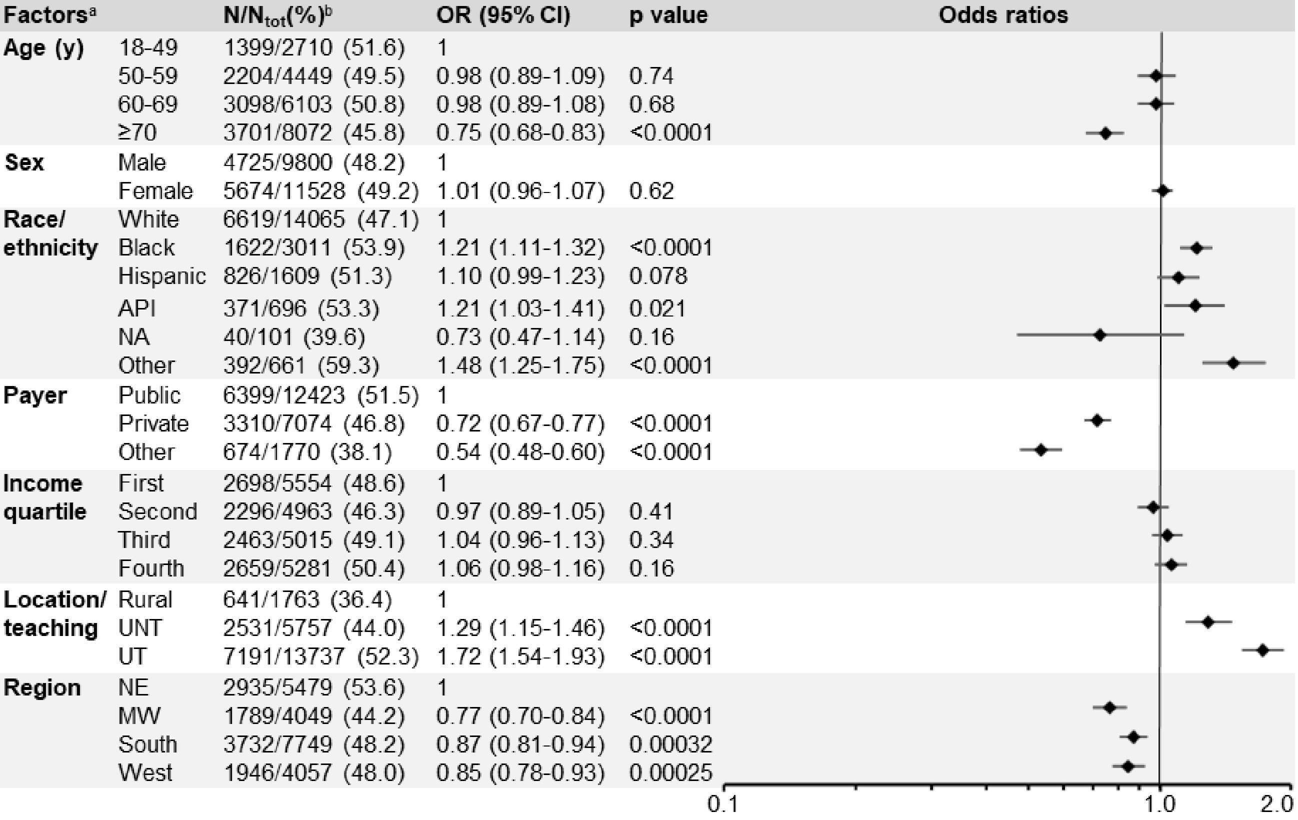

[a] Abbreviations: White = White, non-Hispanic; Black = Black, non-Hispanic; API = Asian/Pacific Islander; NA = Native American; public = Medicare or Medicaid; UNT = urban non-teaching; UT = urban teaching; NE = Northeast; MW = Midwest.
[b] N_{tot} for subgroups differ as missing values were excluded case-wise from analysis.

© 2021 Deeb S et al. *JAMA Network Open*.

eReferences

1. Healthcare Cost and Utilization Project, AHRQ, *Overview of the National (Nationwide) Inpatient Sample (NIS)*. Published 2019. Accessed July 6 2020. https://www.hcup-us.ahrq.gov/nisoverview.jsp.
2. Healthcare Cost and Utilization Project, AHRQ, *NIS Description of Data Elements*. Published 2018. Accessed July 6 2020. https://www.hcup-us.ahrq.gov/db/nation/nis/nisdde.jsp.

© 2021 Deeb S et al. *JAMA Network Open*.
